# Inequality in China’s Food and Nutrition Production and the Decomposition of Contributing Sources

**DOI:** 10.3390/foods14173126

**Published:** 2025-09-06

**Authors:** Wenli Qiang, Jiayi Liu, Baowen Zhang, Die Huang, Yue Xiang

**Affiliations:** College of Earth and Environmental Sciences, Lanzhou University, Lanzhou 730000, China; ljy2023@lzu.edu.cn (J.L.); zhbaowen2024@lzu.edu.cn (B.Z.); hdie2024@lzu.edu.cn (D.H.); xiangy2024@lzu.edu.cn (Y.X.)

**Keywords:** food and nutrition production, inequality, Theil index, Lorenz curve, China

## Abstract

Food and nutrition production play a pivotal role in China’s transition toward a nutrition-sensitive food system. Alongside rapid urbanization and dietary shifts, substantial transformations have occurred in food production patterns. This study investigates inequality in China’s food and nutrition sectors from 1991 to 2021 by employing the Theil index and Gini coefficient, analyzing its drivers from both regional and categorical perspectives. The findings reveal significant disparities in food production concentration across different categories, with notable shifts over the study period. Land-intensive agricultural products—including cereals, oil crops, sugar crops, pulses, roots, tubers, and livestock—exhibited increasing inequality, as indicated by rising Gini coefficients and Theil indices, suggesting greater spatial concentration. In contrast, labor-intensive categories such as fruits and aquatic products showed declining inequality, reflecting broader distribution. Notably, inequality within specific food types (e.g., wheat, beet, and rapeseed production) exceeded disparities among broader food categories. Nutrition inequality, measured by both indices, also increased between 1991 and 2021. However, variations across different nutrients were relatively minor, as diversified nutrition sources mitigated inequality within food categories. Geospatial analysis further highlighted distinct patterns: cereals were the primary contributors to disparities in energy, protein, and mineral supply; oil crops and livestock products drove fat inequality; while vegetables and fruits predominantly influenced vitamin inequality. These findings offer critical insights for optimizing China’s food and nutrition distribution strategies, supporting more equitable and sustainable food system development.

## 1. Introduction

Food security is a critical component of sustainable development in China. Rapid urbanization, economic growth, and rising incomes have driven a marked shift in dietary patterns, with plant-based consumption increasingly supplanted by animal-based products [1,2]. This transition poses a major challenge to meet the demand for the nutritional needs, as food production expansion is constrained by limited cropland and water resources [3,4]. Furthermore, persistent structural inefficiencies, particularly smallholder-dominated and geographically fragmented agricultural operations, have resulted in significant yield gaps relative to production potential. Critically, the spatial dissociation between regional food production and nutritional demand not only generates substantial environmental impacts through extended supply chains but also perpetuates suboptimal dietary patterns that compromise nutritional security [5]. Addressing these systemic challenges necessitates the comprehensive restructuring of production systems and strategic regional specialization in high-value, nutrition-sensitive agricultural commodities [6,7].

The aim of food production is not only to supply sufficient calories to feed people, but also to provide essential vitamins and minerals to maintain human health [8]. National nutrition and health status is an important indicator of a country’s sustainable development [1]. However, traditional food security mainly focuses on cereal production and energy supply in China [9]. As the food production capacity increases, food diversity and nutrition have gradually received attention [1,10,11]. The agglomeration of crop and animal husbandry can improve the efficiency of food production; however, different food production structures among regions lead to various levels of nutrition output, resulting in a mismatch between food and nutrition production and consumption at the regional scale [12]. With increasing health problems related to incorrect components in the diet, China’s food production needs to transform toward providing nutrition and healthy food with the lower environmental and resource costs for the future [1,13]. Thus, the spatial-temporal patterns of nutrition output require an in-depth understanding.

Existing studies have predominantly examined food specialization at the global scale [14,15]. In the Chinese context, however, research has largely focused on individual food categories or types, offering limited insights into the broader patterns of overall food production. Moreover, few studies have systematically assessed disparities in food production equality—particularly in nutritional output—across provincial and regional scales, or explored their interrelationships. To address these gaps, this study examines the inequality of food and nutrition at provincial and agricultural regional scales, utilizing provincial food commodity production data and highly disaggregated commodity-specific nutrient data. Nearly all types of nutrients were considered to illustrate the nutritional output in China. We quantify the spatial–temporal heterogeneity of food and nutrition inequality and identify the sources of this inequality from both geographical and categorical perspectives.

## 2. Literature Review

The distribution of major food production in China has changed significantly over the past few decades. Previous studies have documented the agglomeration of China’s food production across various classification levels, reaching diverse conclusions.

Crop production in China has exhibited a notable geographical agglomeration trend, becoming more pronounced with increased subdivisions [16]. Key to China’s food security, the major production area of cereal crops has shrunk and become concentrated in a few provinces, an increasing number of provinces need to import cereals to fulfill their needs. From 2000 to 2014, the production center of cereals shifted towards the northeast, with the spatial agglomeration pattern becoming more concentrated. High agglomeration areas have gradually migrated to northeastern agricultural regions, while low agglomeration areas have moved towards eastern and southern coastal areas [17]. Recently, fruit production, especially related to apples, pears, oranges, and grapes, has concentrated in Western China [18]. Inner Mongolia consolidated its leading position in the production of oil crops (excluding soybeans) between 2000 and 2016 [19].

The degree of geographical agglomeration in China’s animal husbandry sector has also intensified, transitioning from natural agglomeration to spatial restructuring and optimization [20]. The characteristics of agglomeration vary among different livestock categories. The production center for grass-fed livestock has shifted to the northwest, indicating more rapid growth in the north compared to the south, with beef cattle being the primary driver of this growth [21]. Conversely, pork production has shown relatively lower agglomeration [22].

This spatial–temporal change in China’s food production improved food production efficiency, allowing for increased food demands and nutrition diversity to be met [23]. However, this transition also led to some challenges; for instance, the impact of major production areas on national food security has increased, creating a disproportionate allocation of food security obligations to diminishing production bases, aggravating territorial inequalities [9]. Several systemic challenges have also precipitated, including an extended circulation distance, excessive resource depletion [5], environmental pollution threats [24], and spatial mismatches between food production and water resource distribution [25,26].

## 3. Data and Methods

### 3.1. Data

This study utilized two categories of data sets: (1) Food production data, from 1991 to 2021, covering 36 types of foods and their processed products across China’s 31 mainland provinces. The food items are classified into ten categories: cereals, roots and tubers, sugar crops, legumes, oil crops, vegetables, fruits, stimulants, livestock products, and aquatic products, following the United Nations Food and Agriculture Organization’s methodology (Table 1). Provincial food production data were sourced from the National and Provincial Statistical Yearbook, China Rural Statistical Yearbook, and China Agricultural Statistical Yearbook. We focused on food and nutrition production, without considering food loss or production processes. (2) Food nutrition data, calculated by combining food production data with the food nutrition conversion factors for each food type. This includes metrics for calories, proteins, fats, vitamins (vitamins A, B1, B2, B3, B6, B12, C, D, E, and K), and minerals (Ca, P, K, Na, Mg, Fe, Zn, Se, Cu, and Mn). To facilitate comparisons with macronutrients (energy, protein, and fat), this study aggregated the micronutrients—specifically each vitamin and mineral—to analyze their cumulative characteristics. The food nutrition conversion factors were sourced from the Chinese Food Composition Table and the FAO’s Food Nutrition Table.

### 3.2. Methods

#### 3.2.1. Calculation of Nutrition Production

Nutrition production is calculated as follows:

(1) $$NUTR\;=\;\sum_{i\;=\;1}^{n}\left(P_{i}\cdot c_{i}\cdot EP\right)$$

where *NUTR* is the production of nutrition (calories, proteins, fats, vitamins, and minerals); *P_i_* presents the production of food category *i*; *c_i_* stands for the nutrition conversion factor of food category *i*; and *EP* is the proportion of edible parts for each food category.

#### 3.2.2. Calculation of Concentration and Inequality Indexes

In this paper, we utilized the Lorenz curve to determine the characteristics of China’s food and nutrition production; the cumulative population percentage at the provincial scale was used as the horizontal axis, and the cumulative percentage of food or nutrient production of each province was used as the vertical axis to draw the Lorenz curve of food production inequality. The Gini coefficient is the area under the Lorenz curve, calculated as follows:

(2) $$G=1-\sum_{i\;=\;1}^{n}\left(P_{i}-P_{i-1})(Y_{i}+Y_{i\;-1}\right)$$

where *P_i_* presents the cumulative percentage of the population in each province; *Y_i_* represents the cumulative percentage of food or nutrient production in each province; and *n* is the number of provinces.

The Theil index has become a common statistical method in regional disparity research [27,28,29]. In this study, we use the Theil index to measure the differential characteristics of per capita food and nutrition production among provinces. The formula for this calculation is as follows:

(3) $$T=\sum_{i\;=1}^{n}\;p_{i}ln\left(N_{u}/N_{i}\right)$$

where T is the Theil index; *N_i_* refers to the quantity of food or nutrients produced per capita in province *i*; *p_i_* is the proportion of the population in province *i* of the total; *N_u_* is food per capita or nutrition production nationwide; and *n* is the number of provinces.

#### 3.2.3. The Decomposition of Inequality of Food and Nutrition

(1) Decomposition by geographical group

**Figure 1:**
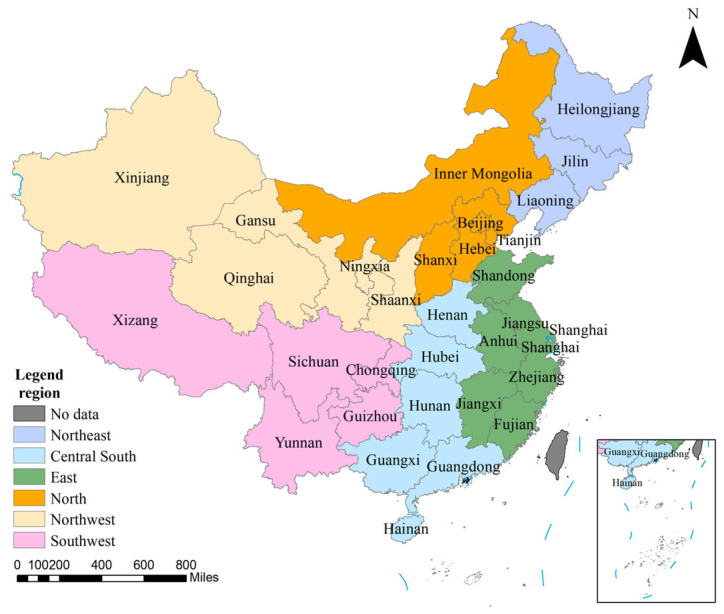
The distribution of provinces and regions. In this study, 31 provinces in China are divided into 6 regions, namely North, East, South Central, Southwest, Northwest, and Northeast China.

The Theil index could be broken down across subgroups defined by geographic or economic criteria, as demonstrated in previous research; thereby, inequality could be divided into between-group and within-group components [30]. Thus, the Theil index was partitioned by region group as follows:

(4) $$T\;=\sum_{g\;=1}^{G}P_{g}T_{g}+\sum_{g\;=1}^{G}P_{g}ln\left(N_{u}/N_{g}\right)$$

where T presents the Theil index of food or nutrition production nationwide, and *P_g_* is the proportion of food or nutrition production for group *g* in the nationwide total. *T_g_* is the Theil index of food or nutrition production within group *g*. *N_u_* indicates the average food and nutrition production per capita nationwide, and *N_g_* is the average food and nutrition production per capita in group *g*. The regional groups are shown in Figure 1.

(2) Decomposition by food categories

The inequality measurement can also be divided into different food sources. The formula for breaking down the Chinese Nutrition Thiel Index based on food sources in this study is as follows:

(5) $$T_{k}=\sum p_{i}w_{ik}\ln(N_{uk}/N_{ik})$$

(6) $$w_{ik}=[(N_{ik}-N_{uk})/(\ln N_{ik}\;-\ln N_{uk})]/\left[(N_{i}-{\;N}_{u}\right)/{(\ln N}_{i}-\ln N_{u})]$$

where *T_k_* represents the Theil index of nutritions produced by food category *k* and *p_i_* denotes the population proportion of province *i* relative to the national total. *N_uk_* represents the national amount of nutrients produced per capita by food category *k*, while *N_ik_* denotes the nutrient output per capita from food category *k* in province *i*. *w_ik_* refers to the weight of nutrients produced by food category *k* relative to the total nutrients from food production; *N_i_* is the nutrient output per capita from all food categories in province *i*; and *N_u_* represents the national nutrient output per capita from all food categories.

## 4. Results

### 4.1. The Centralization of Food and Nutrient Production

**Figure 2:**
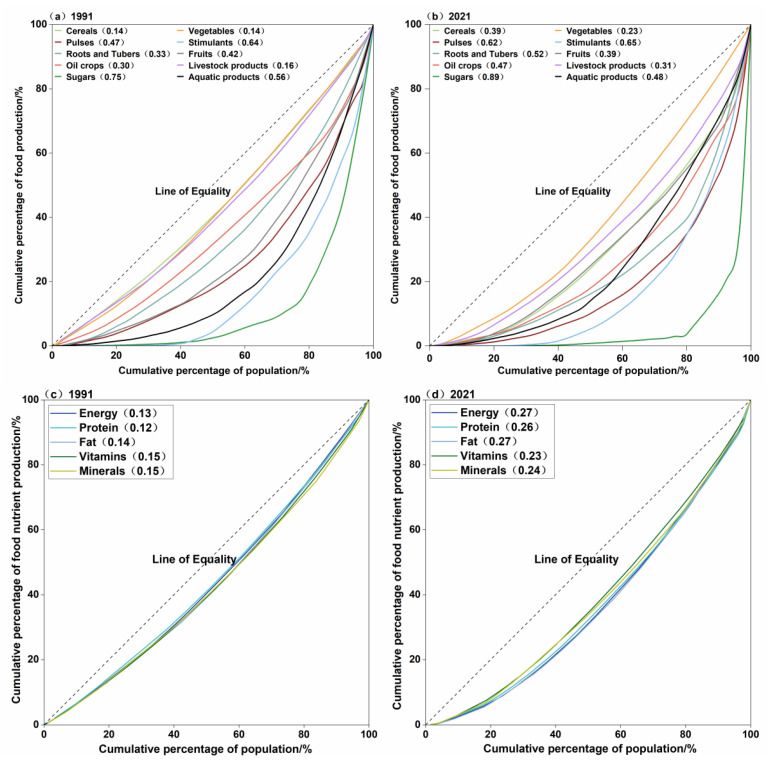
The Lorenz curve of China’s food and nutrition production. The numbers in parentheses in the legend are the Gini coefficients calculated from each Lorentz curve. (**a**,**b**) are the Lorentz curves of the top ten food groups in 1991 and 2021, respectively; (**c**,**d**) are the Lorentz curves of the five major categories of nutrients in 1991 and 2021, respectively.

The concentration of food production has displayed varying characteristics across different categories and underwent significant changes from 1991 to 2021 (Figure 2a,b). Specifically, the concentration of cereals, oil crops, sugar crops, pulses, roots and tubers, as well as livestock products, increased during this period. Conversely, the concentration of vegetables, fruits, and aquatic products decreased, achieving a relative equilibrium with the population distribution. The change in the concentration of stimulant production was comparatively modest.

Sugar production exhibited the highest concentration in 1991, with the top three provinces (Guangdong, Guangxi, and Yunnan) accounting for 61.6% of total production. This concentration further intensified by 2021, as Guangxi’s sugar production increased by 300%, raising its share from 23.6% to 64.3%. Consequently, the combined production share of the top three provinces escalated from 61.6% to 89.5%. Stimulants held the second-highest production concentration throughout the study period. In 2021, the production of stimulants was concentrated in the top five provinces (Yunnan, Fujian, Hubei, Sichuan, and Hunan), collectively representing 64.1% of total production while comprising 21.1% of the population. Pulses emerged as the third largest concentration, surpassing aquatic products, with the top four provinces (Yunnan, Sichuan, Gansu, and Chongqing) contributing 51.0% of the total production in 2021, while only representing 13.3% of the population. The concentration of root and tuber production significantly increased; in 1991, 50.0% of the production originated from provinces with 29.5% of the population, whereas by 2021, this proportion decreased to 16.0%.

Cereals exhibited the lowest concentration levels in 1991, followed by vegetables and livestock products. By 2021, vegetables had the lowest concentration, with cereals falling to fourth position. This shift was largely attributable to the significant increase in Heilongjiang province’s share in cereal production, which rose from 5.0% to 11.2% between 1991 and 2021. The concentration of oil crops also declined, from fourth to fifth place in overall production, driven by decreased production in Shandong province and an increased production share in Inner Mongolia. The relative positions of pulses, roots, and tubers decreased between 1991 and 2021, indicating the higher aggregation of production in these categories. Conversely, the production of aquatic products was more evenly distributed relative to population distribution, moving from eighth to sixth place in terms of overall production during the same period, primarily due to increased production shares in populous provinces such as Hubei and Sichuan.

Compared to food production, the distribution of nutritional output was relatively balanced in 1991, with minimal inter-provincial disparities (Figure 2c). The Gini coefficient ranged from 0.12 to 0.15, indicating low concentrations for energy and protein outputs. However, during the study period, the trend shifted towards an imbalance, and heterogeneity among different nutrients intensified. Provinces with large populations, such as Sichuan, Henan, Shandong, Jiangsu, and Guangdong, which accounted for 9.4%, 7.6%, 7.4%, 5.9%, and 5.6% of the national population, respectively, also held significant shares in energy output, at 10.3%, 6.2%, 7.5%, 7.8%, and 5.7%, respectively. Other nutrients exhibited similar distribution patterns in 1991.

By 2021, the alignment between population distribution and nutritional output was disproportionate, as evidenced by the Lorenz curves for all five nutrients deviating further from the line of equitable distribution (Figure 2d). Energy production exhibited the highest concentration, whereas vitamins had the lowest. Notably, there was a significant decline in nutritional output in some highly populous provinces such as Guangdong. In contrast, provinces with relatively smaller populations, such as Guangxi and Xinjiang, which represented 3.6% and 1.8% of the total population, respectively, increased their shares of vitamin production from 2.8% and 1.9% to 5.7% and 5.3% during the study period. Heilongjiang also increased its energy production share from 3.9% in 1991 to 7.8% in 2021, despite its share in the population decreasing from 3.1% to 2.2%.

### 4.2. The Inequality of Food and Nutrition Production

**Figure 3:**
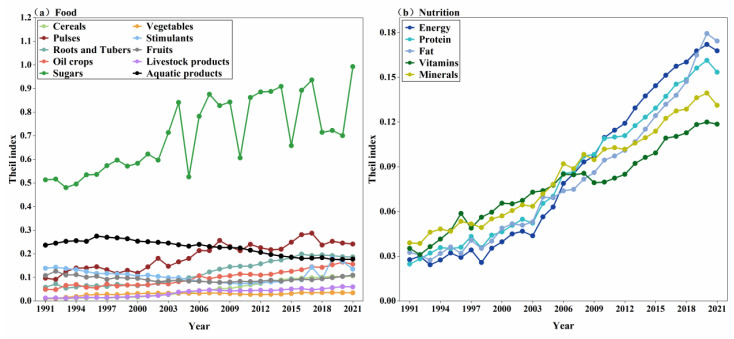
The inequality of food and nutrition production per capita during 1991–2021. (**a**) The Theil coefficient of the ten major categories of food; (**b**) the Theil coefficient of the five major categories of nutrients.

The inequality of food and nutrition output per capita varied during the study period (Figure 3a). The Theil index of stimulants and aquatic products displayed a decreasing trend, while all other categories tended to increase during 1991 and 2021. Cereals had the lowest Theil index in 1991, indicating minor disparity between provinces associated with cereal production per capita. The highest value was attributed to Jilin province with 714 kg of production per capita, and the minimum value was related to Shanghai province with 178.8 kg of production per capita. While the largest value rose to 2273 kg per capita in Heilongjiang, and the smallest value reduced to 16 kg per capita in Beijing, the cereal index showed an increased trend from 0.010 in 1991 to 0.106 in 2021, dropping to the third lowest place in overall production in 2021. The lowest Theil index was observed in vegetables in 2021, followed by livestock products. This is because the centralization trend of vegetables and livestock production is relatively lower than in other categories.

Sugar has the highest inequality, with a much higher Theil index compared to other categories; it displayed a fluctuating increasing trend, specifically between 2003 and 2018, predominately caused by oscillations in yield in the main producing areas. The highest per capita production was found in Hainan, with 598 kg per capita in 1991, while in 2021, the largest value of sugar production per capita increased to 1462 kg in Guangxi; the minimum was 0 across the study period, indicating an expanding gap between provinces. Aquatic production ranked had the second highest levels of inequality in 1991; as its Theil index decreased between 1991 and 2021, mainly due to the rapid development of inland freshwater aquaculture industry provinces. Pulses, roots, and tubers experienced gradual concentration in a few provinces; while most other categories decreased their production, the Theil index of these two nutrients increased dramatically and had the second and third highest levels of inequality in 2021.

Inequality in the production of all nutritional categories exhibited an increasing trend between 1991 and 2021 (Figure 3b). In 1991, the Theil index differences among various nutrients were relatively small, but these differences expanded by 2021. The highest Theil index in 1991 was observed for mineral production, which correlated with cereal production, as the primary source of mineral output at that time. Vitamins and fat ranked second and third, respectively, while energy and protein exhibited the lowest Theil indices. By 2021, fat production had the highest Theil index, followed by energy and protein. The lowest Theil index was observed for vitamin production, predominantly derived from vegetables and fruits, which showed only a slight increase in their Theil indices. Minerals ranked second lowest in terms of the Theil index in 2021, attributable to their derivation from vegetables and livestock products, both of which exhibited lower levels of inequality.

**Figure 4:**
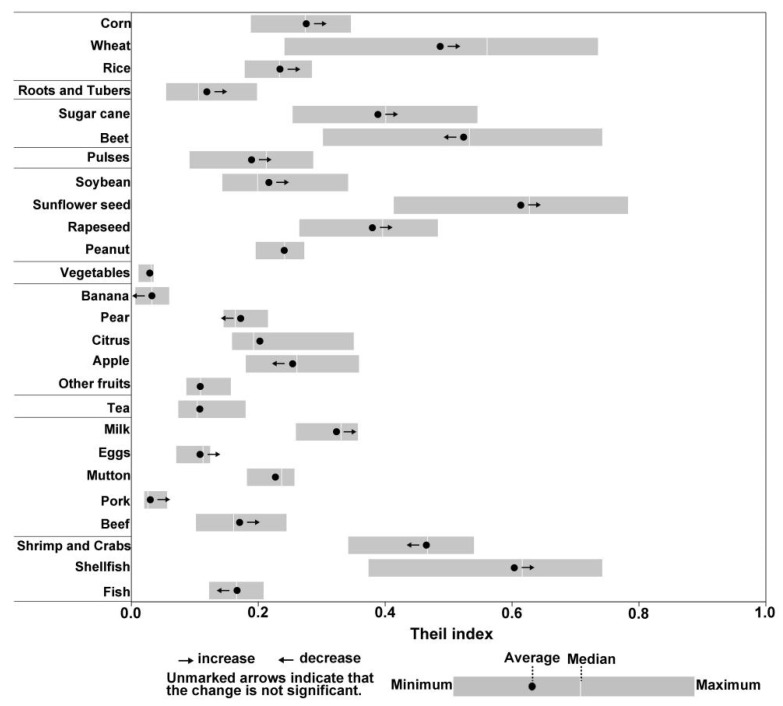
Theil index of China’s food production per capita during 1991–2021. The width of each bar represents the range of Theil indexes observed over the study period.

The coefficient of variation for the per capita yield of different crops exhibits significantly higher variations compared to other categories (Figure 4). The highest average value was observed in sunflower seed, mainly due to its concentrated production in Inner Mongolia, which strengthened during the study period, generating a greater output per capita than other provinces. The Theil index of shellfish, sugar beet, and wheat was also relatively higher than others. The largest value of per capita shellfish production was observed in the Fujian, Liaoning, and Shandong provinces. Sugar beet had the largest per capita production in Inner Mongolia, and Xinjiang. Wheat inequality had the largest max–min value, followed by sugar beet, sunflower seed, and shellfish, which ranged between 0.3 and 0.5. The lowest Theil index was observed in pork production, followed by bananas, tea, eggs, and beef, indicating the relative equilibrium for the per capita output of these categories in production regions.

### 4.3. The Decomposition of Inequality of Food and Nutrition Production

**Figure 5:**
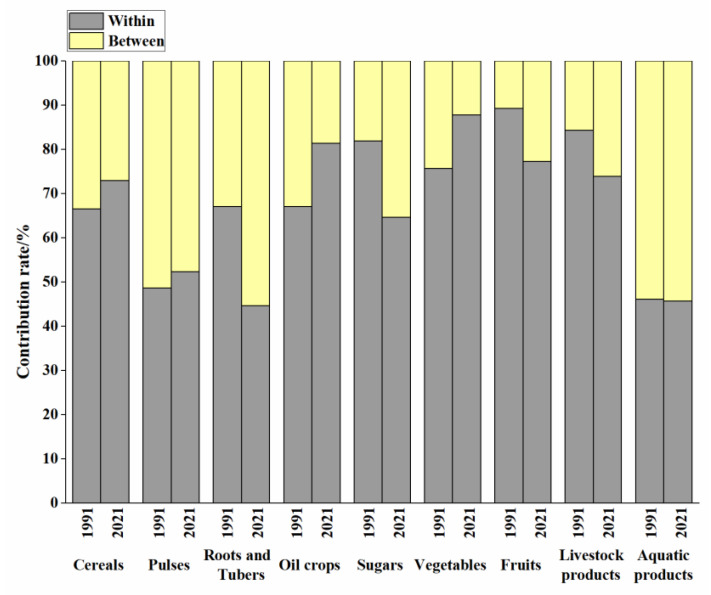
The decomposition of food inequality by regional groups in 1991 and 2021. “Between” represents the contribution of inequality from different regions, while “within” denotes the share of each region’s internal inequality in the region’s total.

The sources of inequality in different food categories in China were analyzed based on geographical regions (Figure 5). The results indicate that the inequality in nearly all types of food production was primarily attributable to differences within regional groups, highlighting the impact of the geographical dispersion of these food categories. However, the contribution of within-group differences varied among categories. The production disparities within groups increased for cereals, pulses, oil crops, and vegetables, indicating a higher concentration of these food categories in major provinces. Conversely, the share of within-group differences for roots and tubers, sugars, fruits, and livestock products decreased from 1991 to 2021, suggesting a trend towards geographic aggregation in their production. The contribution of between-group differences played a major role in aquatic production inequality, mainly due to the necessity for proximity to water bodies.

The primary contributor to nutritional imbalances was within internal groups, the share of which increased from 1991 to 2021 (Table 2). This indicates that inequality in China’s food nutrition primarily stemmed from disparities within regions rather than among them, with nutritional production concentrated in specific provinces. In 1991, the within-group contribution to protein production was the largest. The between-group contribution was relatively higher for vitamin production compared to other nutrients. The internal provinces of East China exhibited the highest disparity in the production of protein, vitamins, and minerals, whereas the internal provinces of Northwest China contributed the least to the production of energy, protein, fat, and minerals. By 2021, within-group differences contributed the most to mineral production inequality, predominantly driven by the internal regions of East, North, and South-Central China. The disparity within these regions showed an increasing trend, while the gap between the Southwest and Northeast regions decreased.

**Figure 6:**
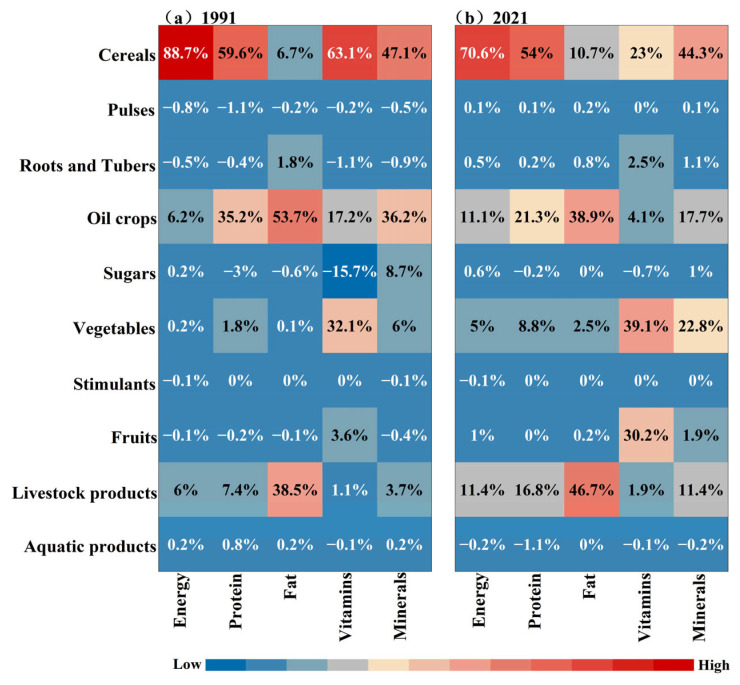
The contribution of different food sources to the inequality of provincial-level nutrition output in China in (**a**) 1991 and (**b**) 2021. Each row represents a different food category, and each column represents a different type of nutrition. The value of the grid stands for the share of each food category in China’s relative nutrition output.

Disparities in China’s food nutrition production inequality were also evident across different food categories (Figure 6). Cereals were initially the major contributor of energy inequality, but their share in energy production decreased during the study period from 88.7% to 70.6%. However, cereals were still a major contributor to the Theil index’s energy increase, followed by livestock products and oil crops. Cereals and oil crops ranked as the top two largest contributors of protein inequality, but their contributions decreased during 1991 to 2021. The share of livestock in protein inequality increased drastically from 7.4% to 16.8%. Oil crops were the largest sources of fat inequality, and also a major contributor to the Theil index’s increase from 1991 to 2001, contributing the largest proportion to fat inequality.

The inequality of vitamins was mainly derived from cereals, with a share that decreased from 63.1% in 1991 to 23.0% in 2021. By contrast, the contribution of fruits increased dramatically, from 3.6% in 1991 to 30.2% in 2021. The proportion of vegetables also experienced a gradual increase and contributed the most to vitamin production inequality. The largest contributor of mineral inequality was cereals during the study period, with a share of 40.3% on average. The share of vegetables increased as the second largest contributor in 2021, while the proportion of oil crops fell from 36.2% in 1991 to 17.7% in 2021. The share of livestock products increased in its contribution to mineral inequality from 3.7% to 11.4%.

## 5. Discussion

### 5.1. Links Between Food and Nutrition Production Inequality

Similarities and disparities were evident in the inequality of food and nutrition during the study period, demonstrating an overall increasing trend. However, inequality in nutrition was relatively lower than that of food, primarily because nutritional elements were dispersed across different food categories, particularly vitamins and minerals, which were predominantly derived from vegetables and cereals. Nonetheless, due to the more concentrated distribution of different food categories in more affluent areas, the inequality in all nutritional categories increased from 1991 to 2021.

The changing trend in nutrition production inequality reflects the transformation of China’s food system. In 1991, the primary role of food production was to meet basic energy supply demands, with grain production being the top priority at the provincial level [31]. Consequently, cereals exhibited the highest levels of equality, as major sources of energy and protein, with these two nutrients exhibiting the lowest levels of inequality in 1991. However, with economic development and improvements in agricultural production capacity, food requirements and supply in China shifted towards diversification and nutritional quality. This shift led to a rapid increase in the production of vegetables, fruits, livestock products, and aquatic products in most provinces, which are major sources of minerals and vitamins. As a result, the production of minerals and vitamins became more balanced by 2021. Conversely, as the importance of cereal production declined at the provincial level, the inequality in energy and protein production increased.

### 5.2. The Driving Factors Behind Changes in Food and Nutrition Production Inequality

The inequality trends in China’s food and nutrition result from the combined effects of natural resources, agricultural technology, policy, and market dynamics. Specific spatial clustering patterns are determined by the alignment between the natural conditions of different regions and the production requirements of various crops [16]. For instance, cereal crops are more suited to mechanized operations. Thus, the cereals production is concentrated in plain regions such as the Sanjiang Plain, Sunneng Plain in Northeast China, Huanghuaihai Plain, and Hetao Plain in North China. Fruits, often planted in hilly or mountainous areas, are primarily concentrated in Guangxi, Shaanxi, and Shandong provinces. Climate change has significantly altered the spatiotemporal patterns of agricultural production in China. A prominent manifestation of this shift is the northward expansion of rice cultivation into northeastern provinces, driven by rising temperatures. This phenomenon mirrors similar observed trends in cereal production expansion across northern Russia, suggesting a broader climate-driven redistribution of agricultural zones at higher latitudes [32].

Additionally, policy directions and the socio-economic status of regions play significant roles in the transformation of China’s food and nutrition production. Decreased food production in Southern China is mainly attributed to urbanization. The disparity between low agricultural income and rising urban wage levels drives labor migration, and the small scale of farms makes mechanization costs prohibitively high, leading to cropland abandonment and reduced cultivation intensity. From 2000 to 2015, regions of urban expansion, particularly in the middle and lower reaches of the Yangtze River agricultural region and southeast coastal areas, experienced significant declines in both farmland and grain production [33,34].

The development of agricultural technology has driven increases in food production. The greater input of machinery and fertilizers has increased yields of the three main cereal crops since 1980 [35]. Yield increases and improved land productivity are major contributors to the rise in food production per capita. Additionally, globalization has influenced China’s food production patterns. Since China’s accession to the WTO, soybean imports have soared while domestic production has declined [35]. Major soybean-producing regions such as Heilongjiang and Jilin in Northeast China have shifted to rice and maize production, increasing the cereal output share of these provinces [36]. Furthermore, recent increases in China’s vegetable and fruit exports have promoted higher production in certain provinces.

With the improvement of living standards and increased health awareness, food consumption in China is shifting from “eating enough” to “eating well” [37]. This shift reflects a dietary transition from primarily plant-based foods to diets with higher reliance on animal proteins, accelerating increases in livestock and aquatic production. Animal foods are more concentrated in populous provinces such as Shandong, Henan, and Sichuan. Agricultural policy also plays a crucial role in food aggregation. Under government guidance, Shouguang in Shandong province has established the largest vegetable wholesale market in the country, becoming a key hub both regionally and nationally. These findings align with patterns observed in other emerging economies. For instance, India’s soybean oil production shows similar geographic concentration in densely populated regions [38], while Brazil’s soybean production expansion has been primarily export-driven. These parallel cases suggest that agricultural concentration in developing economies often follows either domestic demand patterns (as in India) or international market forces (as in Brazil) [39].

### 5.3. Policy Implications

Comprehensive measures are urgently needed to simultaneously ensure food security, nutritional adequacy, resource efficiency, and environmental sustainability. First, optimizing crop redistribution at the county level could help meet China’s nutritional demands while minimizing agricultural inputs (e.g., fertilizers and irrigation water) and reducing environmental impacts [40]. The spatial agglomeration of food production has weakened comparative efficiency advantages for certain crops. Notably, the northward shift in staple crop production to regions with poorer natural endowments introduces significant uncertainties regarding both production stability and ecological risks [9]. This concern is particularly acute for marginal and degraded lands being converted to cultivation in northern regions [41,42]. The growing spatial mismatch between provincial grain production and consumption has exacerbated soil erosion on croplands [2,43] and increased transportation-related carbon emissions by over 60% [44,45]. Strategic policy interventions should leverage existing comparative advantages, such as promoting soybean cultivation in southern China where planting efficiency remains high. Regional crop switching initiatives have already demonstrated dual benefits, simultaneously enhancing environmental sustainability and increasing farmer incomes [46].

Second, enhancing production efficiency in China’s major agricultural zones is crucial for safeguarding national food security. While spatial concentration of food production offers some economic advantages, it simultaneously increases systemic risks by reducing regional supply diversity. Substantial evidence demonstrates that production diversity positively correlates with dietary diversity and improved nutritional outcomes, particularly in developing contexts [47]. To address these challenges, a multi-pronged approach is necessary: First, targeted investments in farmland infrastructure are essential to stabilize and enhance grain yields [48]. Second, rigorous protection of existing arable land must be implemented, particularly in densely populated regions, to maintain both the quantity and quality of productive farmland while curbing excessive conversion for urban and industrial uses [49,50]. Third, comprehensive ecological restoration measures should be prioritized in North China’s agricultural lands to combat soil degradation and ensure long-term productivity.

Third, establishing an efficient food distribution network is critical for ensuring food and nutrition security across China. The current spatial mismatch between production and consumption has forced densely populated urban centers—including Beijing, Shanghai, and Guangzhou—to become heavily dependent on external food supplies. For perishable commodities like vegetables, long-distance transportation from regions as remote as Northeast China exacerbates logistical challenges and environmental costs. To address these issues, policymakers must prioritize optimizing distribution efficiency through targeted interventions. Such measures would not only bolster food security but also mitigate the carbon footprint associated with extensive supply chains. Furthermore, a well-designed distribution system could enhance dietary diversity in monoculture-intensive regions, as empirical evidence confirms the strong linkage between production diversity and improved nutritional outcomes [47]. Complementing these efforts, reforms to agricultural subsidy programs—particularly in key production provinces—are essential for maintaining stable output and ensuring sustainable long-term supply reliability.

## 6. Conclusions and Limitations

Food and nutrition security is key to China’s sustainable development. Equality of food and nutrition production is important to optimize food production under security policies. Few studies focus on different food aggregation categories and their related nutrition output. Thus, the food production data and nutrition conversion parameters reveal a trend of inequality in food and nutrition production. The results suggest that the concentration of food production varies across categories and has undergone significant changes from 1991 to 2021. Land-intensive agricultural food production has become more concentrated, while labor-intensive food production is more widely distributed. The inequality of food production was much higher than nutrition, mainly due to the diverse sources of available nutrients, especially vitamins and minerals. The decomposition of inequality shows that the concentration of certain nutrition varies between regions, indicating that food and nutrition disparities exist on a geospatial scale. Cereals are the major source of energy, protein, and minerals inequality; oil crops and livestock products dominated fat inequality; and vegetables and fruits contributed the most to vitamins inequality.

While our findings align with previous research [16,17,21], this study provides novel contributions by (1) systematically analyzing all major food production categories in China, and (2) employing inequality indices to quantify spatial disparities. These methodological advances offer a more comprehensive understanding of China’s food production landscape and complement existing literature. Limitations were identified regarding the spatial change in food production, focusing on one specific category. In addition, our study links and compares food and nutrition production at both regional and provincial scales, effectively illustrating the temporal-spatial changes in nutrition and providing a reference to China’s transformation to a nutrition-sensitive food system. Furthermore, this research aims to inspire further studies on the environmental impacts of food production concentration and emissions regarding disparities between food production and consumption.

This study also has two limitations that need to be addressed in further research. Firstly, data were limited to the provincial scale, which cannot provide details of food and nutrition inequality at the county level, mainly due to the lack of data availability. Second, the inequality of food and nutrition production is influenced by many factors, and our study only qualitatively discussed the impacts of these factors. Further quantitative studies need to be conducted to identify relationships between factors and fully understand their impact.

## Figures and Tables

**Table 1:** Food categories and types.

Food Category	Food Types
Cereals	Wheat, Rice, Corn, Sorghum, Millet, Others
Pulses	Pulses
Roots and Tubers	Potatoes, Others
Oil crops	Soybean, Linseed, Groundnut, Sunflower seed, Rapeseed, Sesame seed
Sugars	Sugar cane, Sugar beet
Vegetables	Vegetables
Stimulants	Tea
Fruits	Apple, Pear, Date, Grape, Persimmon, Citrus, Banana, Pineapple, Others
Livestock products	Pork, Mutton, Beef, Poultry, Milk, Eggs, Bee honey
Aquatic products	Fish, Shellfish, Shrimp, Crabs

**Table 2:** Contribution of between and within geographical regions to China’s food nutrition inequality (%).

		Energy		Protein		Fat		Vitamins		Minerals	
		1991	2021	1991	2021	1991	2021	1991	2021	1991	2021
Between		30.4	12.0	21.3	12.5	24.1	15.6	34.6	14.4	31.3	9.8
Within	North	7.5	24.2	7.8	25.4	10.8	25.6	4.7	23.8	5.1	27.5
	Northeast	7.3	7.2	17.6	6.9	6.4	1.6	3.4	1.0	11.3	5.6
	East	18.4	31.9	21.0	29.3	7.5	29.8	33.0	33.9	28.8	32.7
	Central South	19.4	22.6	12.7	23.9	27.6	25.0	8.7	18.7	8.4	22.3
	Southwest	11.6	0.8	15.2	0.8	20.8	1.8	10.3	1.1	11.0	0.1
	Northwest	5.5	1.3	4.5	1.3	2.8	0.4	5.4	7.0	4.1	2.0

## Data Availability

The original contributions presented in the study are included in the article, further inquiries can be directed to the corresponding author.
